# Fluorescent Gold Nanoclusters for Selective Detection of Dopamine in Cerebrospinal fluid

**DOI:** 10.1038/srep40298

**Published:** 2017-01-09

**Authors:** Saravanan Govindaraju, Seshadri Reddy Ankireddy, Buddolla Viswanath, Jongsung Kim, Kyusik Yun

**Affiliations:** 1 Department of Bionanotechnology, Gachon University, Gyeonggi-do, 13120, Republic of Korea; 2 Department of Chemical and Biological Engineering, Gachon University, Gyeonggi-do, 13120, Republic of Korea

## Abstract

Since the last two decades, protein conjugated fluorescent gold nanoclusters (NCs) owe much attention in the field of medical and nanobiotechnology due to their excellent photo stability characteristics. In this paper, we reported stable, nontoxic and red fluorescent emission BSA-Au NCs for selective detection of L-dopamine (DA) in cerebrospinal fluid (CSF). The evolution was probed by various instrumental techniques such as UV-vis spectroscopy, High resolution transmission electron microscopy (HTEM), X-ray photoelectron spectroscopy (XPS), X-ray diffraction (XRD), Fourier transform infrared spectroscopy (FTIR), photoluminescence spectroscopy (PL). The synthesised BSA-Au NCs were showing 4–6 nm with high fluorescent ~8% Quantum yield (QY). The fluorescence intensity of BSA-Au NCs was quenched upon the addition of various concentrations of DA via an electron transfer mechanism. The decrease in BSA-Au NCs fluorescence intensity made it possible to determine DA in PBS buffer and the spiked DA in CSF in the linear range from 0 to 10 nM with the limit of detection (LOD) 0.622 and 0.830 nM respectively. Best of our knowledge, as-prepared BSA-Au NCs will gain possible strategy and good platform for biosensor, drug discovery, and rapid disease diagnosis such as Parkinson’s and Alzheimer diseases.

Dopamine (DA) is a neurotransmitter that belongs to catecholamine family and plays important roles in nervous system functions such as motor control, mood, emotions, and memory^1^,^2^,^3^. Therefore, the functions of neurotransmitters are elucidating for the development of pharmacological agents that can help prevent several neurological diseases in humans. Abnormal and insufficient levels of DA were linked to the neurological disorders, and they lead to the development of Parkinson’s and Alzheimer’s disease^4^,^5^. In the neurotransmitter motor process, the concentrations of DA in disease-affected patients were found to be lower than in healthy individuals, decreasing to nM concentrations^6^. DA is metabolized through two major pathways, producing two kinds of acidic products, homovanillic acid (HVA), and 3,4-dihydroxyphenylacetic acid (DOPAC). Generally, human functions are evaluated according to the concentration of HVA in CSF that contains cellular information and biochemicals that are favorable for the brain activation^7^. Purification and maintenance of CSF depends on the efficacy of the clearing mechanism^8^, and the evaluation of DA metabolism in the neurochemical processes is critical.

Noble metal nanomaterials have been developed in various fields. Gold nanoparticles and gold nanoclusters (Au NCs) have received a lot of attention in nanotechnology, compared with other materials^9^. In particular, Au clusters are sub-nanometer and fluorescent, and demonstrate electronic transitions between HOMO-LUMO energy levels^10^. Smaller-sized Au NCs have strong fluorescent emission, which permits the quantification of nucleus-targeting molecules with single-particle sensitivity^11^,^12^. Several methods were used for the synthesis of Au NCs, such as template-based (polymers, proteins, DNA, and dendrimers) and ligand-protected (phosphine ligands and thiolate-capped gold nanoclusters) methods^13^. A “green” synthetic method was developed, where bovine serum albumin (BSA) protein reacted with the Au^3+^ ions, and converted them into Au^+^ ions, with the help of tyrosine, aspartate, glutamate, asparagine, and glutamine presents in the protein^14^. “Green” synthetic protein-protected Au NCs attracted a lot of attention in biomedical research due to their high fluorescence, stability, and reduced toxicity^15^.

With the development of fast and sensitive analytical techniques, several methods such as, chemiluminescence, enzymatic techniques, capillary electrophoresis, electrochemical sensing, and spectrofluorometry have been used for the detection of different compounds^16^. Among them, fluorescence detection methods received special attention compared with other analytical methods because of their sensitivity and selectivity. Fluorescence intensity quenching of the sample refers to the fundamental phenomenon used to collect biochemical information^17^.

Different types of biomarkers are urgently needed for the monitoring and diagnosis of Alzheimer’s and Parkinson’s diseases^18^. In the past two decades, several studies have been conducted in order to allow the detection of DA and other CSF proteins. DA was detected by fluorescence quenching through CdSe quantum dots (QDs). Various concentrations of DA were detected by the quenching of cysteine-capped indium phosphide/zinc sulfide QDs, in the presence of ascorbic acid, and the detection limit was found to be 5 nM^19^. The detection of DA in the biological samples is hindered by some acids, which also presents a challenge for the development of highly sensitive methods. Therefore, for the detection of DA at low concentrations, accuracy and speed of detection are important factors to be considered. The detection of DA in CSF samples by fluorescence quenching of BSA-Au NCs is an important new method, with low cost, high sensitivity, and reduced time consumption.

The focus of this study is the development of one-pot synthesis, environmentally benign, and quantum sized novel fluorescent BSA-Au NCs, which exhibit strong red fluorescence emission at 650 nm. The developed method has many advantages, such as high sensitivity, stability, low cost, and eco-friendliness. We demonstrated that the obtained BSA-Au NCs to detect DA, DA in CSF via quenching of fluorescence through the oxidation of DA into dopamine-O-quinone (DQ). DQ accepts the electron from the BSA-Au NCs, which leads to fluorescence quenching, depending on the concentration of dopamine.

## Experimental Section

### Materials

Gold (III) chloride trihydrate (HAuCl_4_.3H_2_O), sodium hydroxide (NaOH), and L-dopamine hydrochloride Human serum albumin,Glucose, Sodium chloride, potassium chloride, magnesium chloride, and calcium chloride were purchased from Sigma-Aldrich, USA. BSA was purchased from Millipore LEE, South Korea. CSF was obtained from LEE Biosolutions, USA. TEM sample grid, with the following specifications: Ultrathin Carbon Type-A, 400 meshes, copper, approximate 42 μm grid hole size, was purchased from Ted Pella, Inc., USA. All chemicals were used without any further purification.

### Instrumentation and Spectroscopic Analyses of BSA-Au NCs

BSA and as-synthesized BSA-Au NCs were diluted in DI water, and dark field images were taken using PTI UV illuminator, at 365 nm. Highly diluted solution of BSA-Au NCs was used to record the UV-Vis spectrum, using UV-Vis spectra of prepared BSA-Au NCs, obtained by Varian Cary 100 UV-Vis spectrophotometer. HRTEM images were recorded using Tecnai™ G2 F30 Series, The ultimate Nano-Analysis System which was equipped with 300 kV Schottky FEG with high maximum beam current, patented S-TWIN objective lens, 0.17 nm of HR STEM resolution, 0.20 nm of point resolution, and 0.102 nm of line resolution was purchased from Hillsboro, USA. X-ray diffraction (XRD) patterns of the particles were obtained using Rigaku Rint 2200 Series X-ray Automatic Diffractometer (Cu Kα radiation at a wavelength of 1.5406 Å) from Texas, USA. XPS spectra were recorded using Thermo Scientific K-Alpha^TM+^ X-ray Photoelectron Spectrometer System equipped with 100–4000 eV range of motion, 180° double focusing hemispherical analyzer 128-channel detector, Al Ka micro-focused as X-ray source, obtained from Thermo Scientific, USA. Fluorescence spectra were recorded with an excitation wavelength of 450 nm, at room temperature, using Photoluminescence (PL) spectra of BSA-Au NCs that were obtained using fluorescence spectrometer (QuantaMaster, Photon Technology International, NJ, USA) equipped with a xenon lamp (Arc Lamp Housing, A-1010B™), monochromator, and power supply (Brytexbox). FTIR spectra were recorded using Bruker vortex high resolution 70 FTIR spectrometer, equipped with BRUKER FT-IR Vertex 70 with a micro plate extension HTS-XT and ATR-units, from Billerica, MA, USA. All fluorescence lifetime decay curves were recorded using Photon Technology International (PTI) EL series of nanosecond pulsed LEDs designed for the EasyLife II with an auto adjustable excitation wavelengths ranging from 260 to 650 nm. Instrument Response Function (IRF) was recorded by using 1% LUDOX^®^ LS colloidal silica suspension water as background signal. The curve fitting data were obtained with using the EasyLife II via a serial RS232 and s USB connection under the control of the program EasyLife II.

### Quantum yield measurements

The as-synthesized BSA-Au NCs (1 mg in 10 mL) solution was directly transferred into a cuvette and the QY was measured using Fluoro-Q2100 Quantum Yield system at 450 nm excitation wavelength, and calculated according to the following expression:


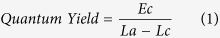


*Ec* represents the emission produced by direct excitation light, *La* is the total amount of excitation light, and *Lc* is the amount of light after the direct excitation^20^.

### Synthesis of BSA-Au NCs

BSA-Au NCs were synthesized by slight modifications of Jianping Xie *et al*.^21^. In a typical synthesis, 5 mL of 10 mM HAuCl_4_ was mixed with 5 mL of BSA (50 mg/mL) solution under magnetic stirring at 800 rpm in the dark chamber. After 30 min, 500 μL of 1 M Sodium hydroxide (NaOH) was added, and heated at 50 °C under vigorous stirring for 3–4 h. This led to the change the color of reaction mixture from light yellow to brown. The color change indicates the starting point of gold nanoclusters formation. The samples were freeze-dried in order to collect pure powder of BSA-Au NCs after synthesis.

### Sensing of DA using BSA-Au NCs

The detection of DA was performed in PBS buffer (0.5 mM, pH 7.2). Approximately 10 mg of BSA-Au NCs were dissolved in 1000 mL of DI water, and sonicated thoroughly for 15 min. After the sonication, this stock solution was used for the detection of DA. In a typical run, 200 μL of 10 mg/L stock solution in 450 μL of PBS buffer, and 50 μL of DA solutions at different concentrations, ranging from 1 to 10 nM were added into a cuvette and incubated for 5 min and PL spectra were recorded at 450 nm wavelength. All PL spectra were recorded at ambient temperature. All measurements were carried out in triplicate for accurate calculations to develop a standard protocol.

### Detection of DA in Biological fluid

The human CSF was obtained from LEE Biosolutions, USA and directly used in the experiment without any further purification as biological fluid. The detection of DA in CSF was followed by the well-established spiked method published elsewhere^22^,^23^ and all PL spectra were recorded at ambient temperature. Prior to the detection, the aliquot (300 μL) of CSF was spiked with standard DA concentrations range from 0–6 nM. In brief, 50 μL of spiked DA solutions at different concentrations were added into a cuvette containing 200 μL of 10 mg/L stock solution in 450 μL of PBS buffer, and incubated for 5 min. After 5 min incubation period, PL spectra were recorded at 450 nm wavelength. All sensitive and selective measurements were carried out in triplicate for accurate calculations to develop a standard protocol.

## Results and Discussion

### Synthesis of BSA-Au NCs

**Figure 1:**
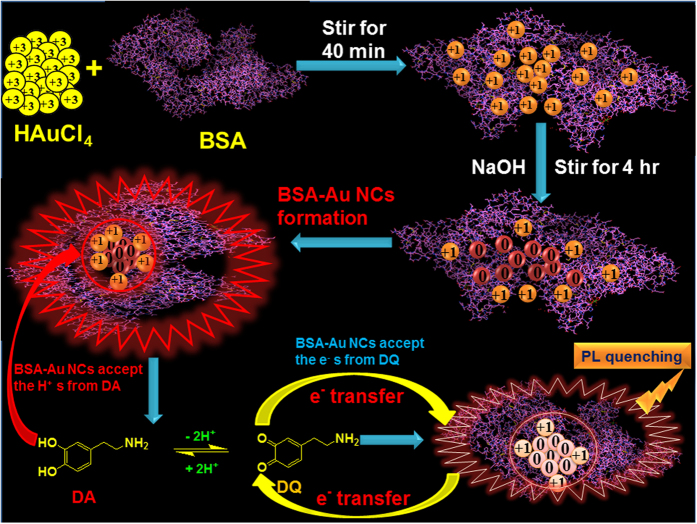
Schematic representation of overall reaction scheme for synthesis of BSA-Au NCs.

In the present work, we have reported the facile synthesis of environmentally benign protein protected BSA-Au NCs for the selective detection of DA in CSF and fluorescence quenching of BSA-Au NCs in presence of DA as illustrated in Fig. 1. An aqueous solution containing HAuCl_4_ and BSA were mixed thoroughly at room temperature until light yellow reaction mixture turned into dark yellow. Here, vigorous stirring may be attributed to the reduction of Au^3+^ to Au^1+^ of most of the gold atoms, due to the reducing and stabilizing activities of BSA native protein. The NaOH solution was added to the reaction and heated while stirring for appropriate time. During this reaction, color change from dark yellow to reddish brown confirmed that the gold atoms were reduced from Au^1+^ to Au^0^ to form clusters^24^. BSA acts as a reducing and stabilizing agent, and performs an important role in the size control of BSA-Au NCs. NaOH increases pH value, which improves the reducing power of the BSA^25^. The concentration of gold atoms increased according to the reaction time. Gold precursor was depleted and reduced after the reducing agent was introduced. Homogeneous nucleation occurred at the supersaturating level of gold atoms, which led to the aggregation of BSA-Au NCs^26^. The increase in nucleation reaction during a relatively short period led to the formation of a large number of small size clusters^21^,^27^. In order to obtain small size of NCs with 8% of high fluorescence QY, a fast reaction was performed by increasing the temperature during the short reaction time. Finally, Au^3+^ ions converted into Au^0^ ions during the short reaction time and formed BSA-Au NCs. These as-synthesized BSA-Au NCs are approximately contains 30 gold atoms per single nanocluster with 8–10% QY^28^.

### Morphological studies

**Figure 2:**
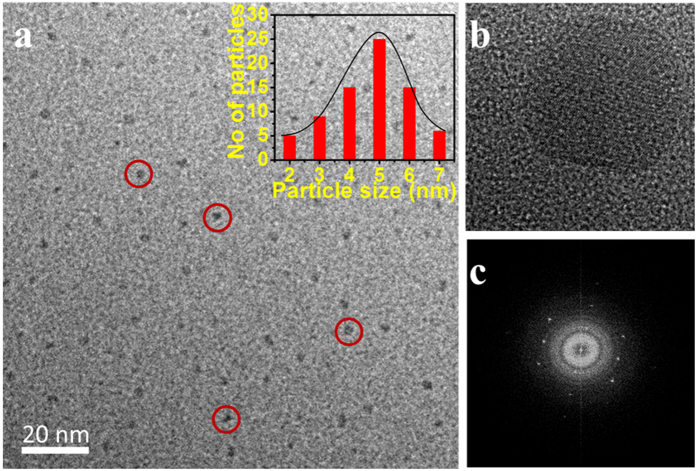
Morphology of BSA-Au NCs (**a**) TEM image, inset image illustrates the average particle size distribution, (**b**) HTEM lattice spacing between faces and (**c**) selected area diffraction SAED pattern of BSA-Au NCs.

TEM image demonstrates that average sizes of BSA-Au NCs were 4–6 nm, and that all clusters were well distributed (Fig. 2a). Figure 2b demonstrates the crystallinity of BSA-Au NCs, in the face-centered cubic (*fcc*) of Au. Lattice fringe spacing could correspond to the (111) reflection of *fcc* of Au. Figure 2c shows the selected area diffraction (SAED) pattern of BSA-Au NCs, indicated that Au nanocrystals were well organized.

### XPS studies

**Figure 3:**
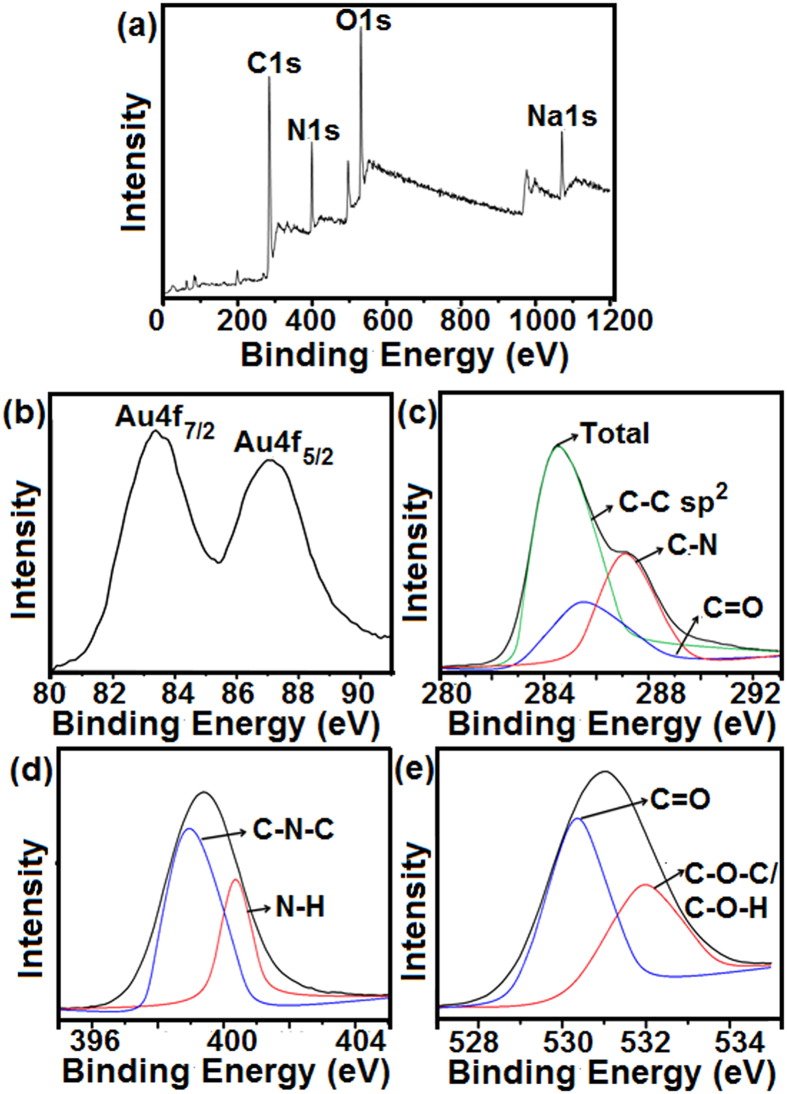
The total SurveyX-Ray photoelectron spectra of as-prepared BSA-Au NCs, (**a**) full range XPS spectrum of C1s, N1s, O1s and Na1s of BSA-Au NCs, individual high resolution XPS spectra of (**b**) Au 4 f, (**c**) C1s, (**d**) N1s and (**e**) (O1s).

The full range BSA-Au NCs XPS spectra depicted in Fig. 3 which illustrates binding energies of all elements, including Au, O, N, and C, in BSA-Au NCs. Elemental peaks of C, N, and O were specifically derived from BSA protein and Au peak was derived from the Au nanocluster. In the full range spectrum, binding energies at 285 (Fig. 3c), 399 (Fig. 3d), and 531 eV (Fig. 3e) were attributed to C1s, N1s, and O1s, respectively. The binding energy at 1090 eV was assigned to Na1s, and this peak was increased following the addition of NaOH to the reaction mixture. XPS spectrum of Au 4 f is depicted in Fig. 3a. The oxidation state of gold atoms in the BSA-Au NCs was determined by X-ray photoelectron spectroscopy (XPS). Initially, the oxidation state of gold (Au) atom in HAuCl_4_ was Au^3+^ and the majority of gold (Au) atoms were reduced from Au^3+^ to Au^1+^ when BSA protein was added to the HAuCl_4_ solution. BSA-Au NCs were formed after the addition of 1 M NaOH solution to the reaction mixture, and finally most of the inner core gold atoms were reduced from Au^1+^ to Au^0^^29^. Several outer sphere gold atoms had Au^1+^ oxidation state, because some of the carbonyl functional groups containing amino acids from BSA native protein were coordinated with the outer sphere gold atoms during BSA-Au NCs formation. BSA-Au NCs were also stabilized with a thiol functional group (-SH) of cysteine amino acids, which were present in the BSA native protein when disulfide bonds were broken down into thiol functional groups, following the addition of 1 M NaOH solution to the reaction mixture^30^.

The number of gold atoms in BSA-Au NCs was confirmed by XPS (Fig. 3b). Au 4 f binding energy full width at half maximum (FWHM) was determined to be ~0.92 eV, which indicates that BSA-Au NCs were formed with approximately 30 gold atoms, after the addition of 1 M NaOH solution to the reaction mixture^28^. The binding energies of Au 4f_7/2_ and Au 4f_5/2_ were determined to be 83.2 and 86.3 eV, respectively, and this confirms the formation of stable BSA-Au NCs, with most of gold atoms close to an Au^0^ oxidation state. The characteristic binding energies, observed at 284.5, 285.9, and 287.7 eV, were attributed to the C-C sp^2^, C=O, and C-N bonds, respectively. Binding energy peaks observed at 399.2 and 400.85 eV are attributed to the C-N-C and N-H bonds, respectively (Fig. 3d). The XPS spectrum of O1s, and binding energies observed at 530.1 and 532.21 eV are attributed to C=O and C-O-C/C-O-H bonds, respectively (Fig. 3e).

### XRD studies

**Figure 4:**
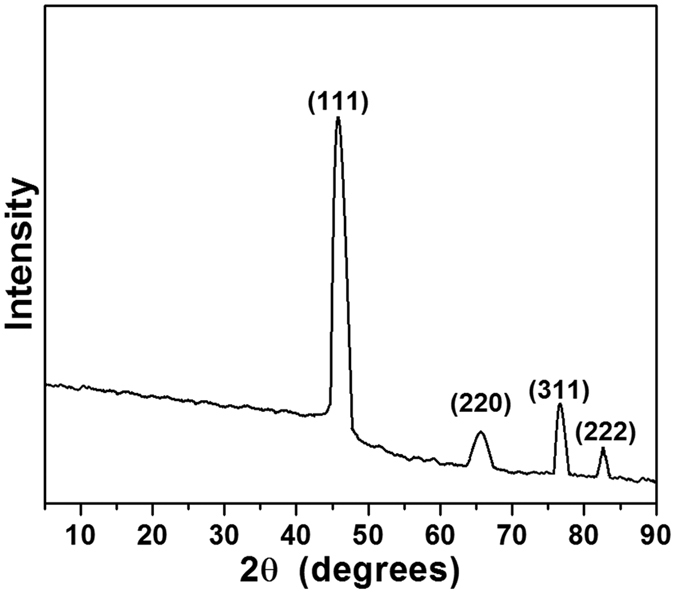
XRD spectrum for BSA-Au NCs.

The as-synthesized BSA-Au NCs crystallinity and diffracted angles were examined using XRD from 5 to 90 degrees (Fig. 4). It shows the distinct 2θ peaks were observed for BSA-Au NCs at 45.5° and 65.6°, 77.89°, and 83.2° corresponding to (111) and (220), (311) and (222) lattice planes, respectively and the peak appeared at 45.5^o^ is more intense than other peaks^31^,^32^. According to HRTEM results, as-prepared BSA-Au NCs were uniformly dispersed and Au has the face-centered unit cell (fcc) in the BSA-Au NCs.

### FTIR studies

**Figure 5:**
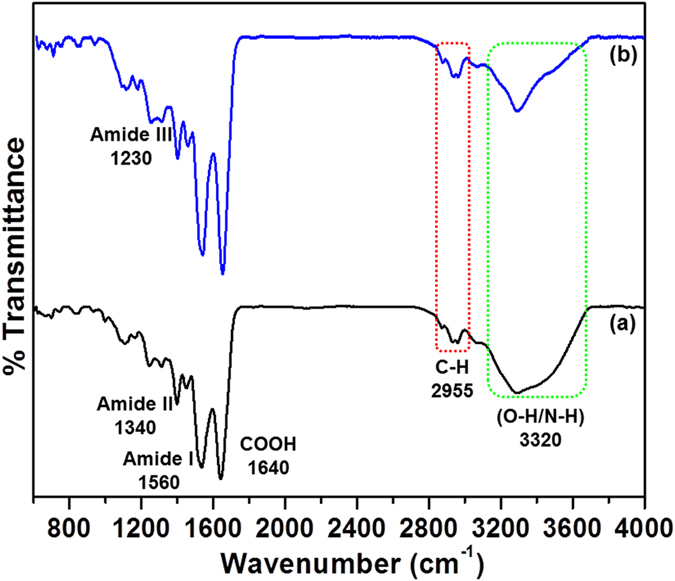
The FTIR spectrum of (**a**) BSA-Au NCs and (**b**) BSA.

The FTIR spectrum of BSA and BSA-Au NCs was illustrated in Fig. 5. The peaks observed at 2955, 3320 cm^−1^ were responsible for C-H and O-H/N-H stretching frequencies respectively in both curves. The characteristic stretching and bending vibrations of amide I, amide II, and amide III were identified at 1560, 1338, and 1230 cm^−1^ respectively, and these peaks were raised due to presence of peptide bonds in BSA native protein as shown in curve b. In the case of BSA-Au NCs, a sharp peak was observed at 1390–1450 cm^−1^ for the assigned secondary structure of β-sheet and this peak can be seen after nanoclusters formation with BSA protein as illustrated in curve a. However, the intensity of curve a, was decreased when compared to the curve b due to conformational changes that were happening after cluster formation^32^.

### Optical properties

#### UV-vis

**Figure 6:**
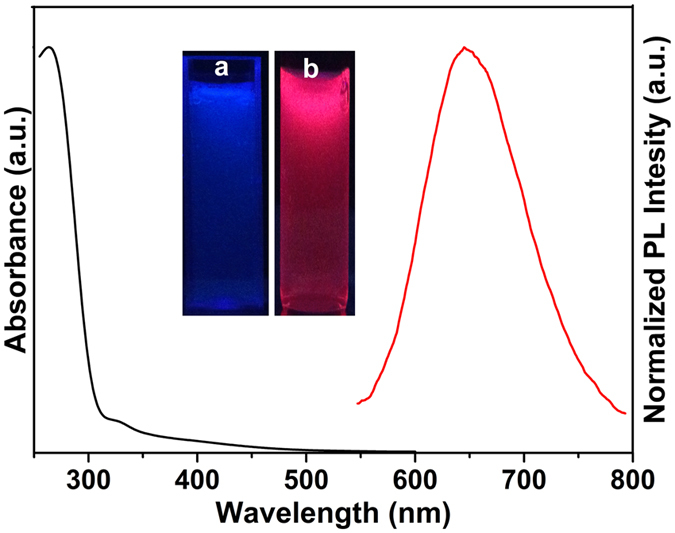
UV-vis and PL intensity absorbance of BSA-Au NCs in PBS buffer and inset image shows the (**a**) BSA and (**b**) BSA-Au NCs under UV illumination.

Considerable UV-vis absorbance peak was not observed due to because of quantum size and functionalization of protein on its surface as shown in black color curve (Fig. 6). The red color curve explains the PL emission peak of BSA-Au NCs at 650 nm when the sample was excited at 450 nm. The inset Fig. 6a shows that normal, pale yellow colored BSA solution emits blue fluorescence under the UV light, whereas after the reaction of BSA with HAuCl4, brown BSA-Au NCs solution emits red fluorescence as shown in Fig. 6b.

#### PL studies

**Figure 7:**
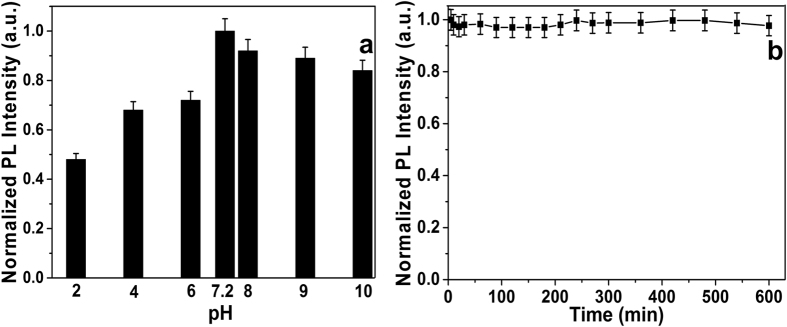
The PL response and stability of BSA-Au NCs as a function of (**a**) pH and (**b**) different time intervals.

Here in this study, we have recorded BSA, BSA-Au NCs fluorescence spectra in PBS buffer, with a strong red fluorescence emission at 650 nm and an excitation wavelength of 450 nm (Fig. 7a). The PL quenching of BSA-Au NCs as a function pH in PBS buffer was illustrated in Fig. 7a. The PL intensity of BSA-Au NCs was quenched ~55% in extreme high acidic medium due to destabilization of protein protected gold nanoclusters via transfer of electrons from CB of BSA-Au NCs to the medium. Whereas in case of extreme basic medium, the significant PL intensity was not quenched due to stabilization of hydroxide ions (HO^−^) and protein protected gold nanoclusters. However, at neutral pH the highest PL intensity was observed. Interestingly, the synthesized BSA-Au NCs were stable at 5–10 pH. Therefore, we have chosen pH 7.2 as an optimum condition for this study. Finally, the stability of the BSA-Au NCs at different time intervals was investigated. The BSA-Au NCs were exhibiting high stability at room temperature for 600 min without elude their original PL intensity (Fig. 7b). Based on these observations, we confirmed that the synthesized NCs have excellent ability to sense the DA at optimal conditions.

#### DA sensing

**Figure 8:**
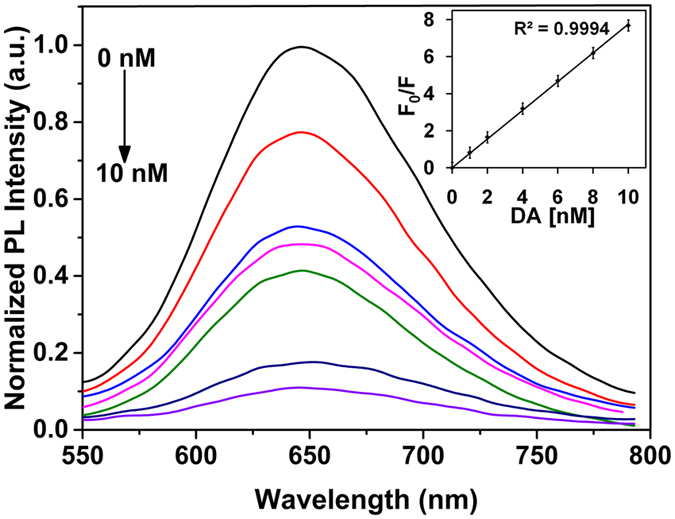
The PL emission spectra of BSA-Au NCs by the addition of various concentrations of DA (λ_ex_ = 450 nm). Inset image shows the dependence of the *F*_*0*_*/F* value on the concentration of DA within the range of 0 to10 nM. Inset figure explains the linear relationship between the *F*_*0*_*/F* value and the concentration of DA within the range of 0 to 10 nM and results were showing a linear fit to the Stern-Volmer equation. The experiment was repeated three times and data was expressed as the mean ± standard deviation.

The BSA-Au NC fluorescence intensity significantly decreased when the addition of DA concentrations increased from 1 to 10 nM (Fig. 8). In this study, BSA-Au NC fluorescence intensity decreased approximately up to 91% in the presence of quencher DA. The relative change of the fluorescence intensity versus the initial intensity (F_0_/F) at 450 nm as a function of DA concentration ([DA]) is presented in inset Fig. 8. The fluorescence quenching mechanism was described by Stern Volmer equation^33^.We have calculated the LOD and found that it was as low as 0.622 nM (calculated at a signal-to-noise ratio of 3). This proves that BSA-Au NCs are a promising, sensitive tool for the detection of DA. The comparison of detection performances of various fluorescent sensors for detection of dopamine was illustrated in Table 1.

#### Real sample analysis

**Figure 9:**
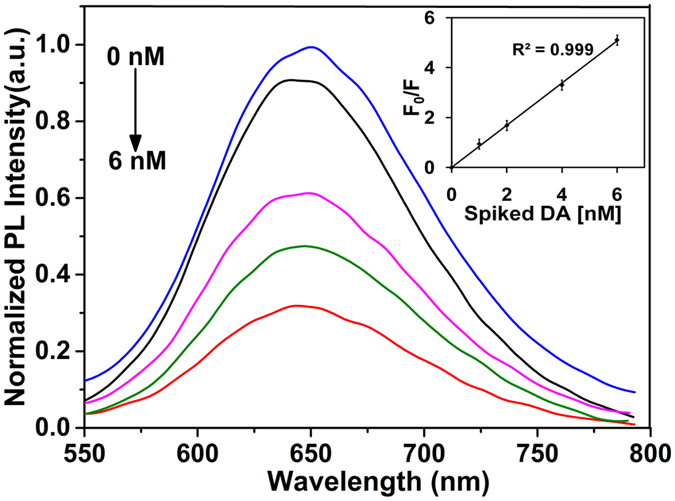
PL spectra of BSA-Au NCs in the presence of different concentrations of spiked DA ranging from 1 to 6 nM and the corresponding response of normalized fluorescence intensity versus concentration of spiked DA. The inset figure displays corresponding response of normalized fluorescence intensity of BSA-Au NCs (*F*_0_/*F*) versus concentration of spiked DA(*F*_0_ and *F*) represent the PL intensity in the absence and presence of DA, respectively and showing a linear fit to the Stern-Volmer equation. The experiment was repeated three times and data was expressed as the mean ± standard deviation.

To investigate the ability of synthesized BSA-Au NCs based biosensor, various concentrations of DA range from 0 to 6 nM were spiked into the CSF and recorded the fluorescence spectra (Fig. 9). Interestingly, the fluorescence intensity of BSA-Au NCs was gradually decreased ~65% upon addition of various concentrations of spiked DA into the CSF. The Fig. 9 shows the relative change of the fluorescence intensity versus the initial intensity (F_0_/F) at 450 nm as a function of spiked DA in CSF concentration ([DA]).The plot of ΔF/F at 450 nm against spiked [DA] in CSF showed linearity in 0 to 6 nM range, with the squared correlation coefficient (R^2^) of 0.999 (inset figure). The LOD was found as low as 0.830 nM (calculated at a signal-to-noise ratio of 3). With these observations we have concluded that BSA-Au NCs have ability to detect DA in biological body fluids. The calculations of spiked and recovery concentrations of DA in CSF were shown in Table 2.

#### Comparison studies

**Figure 10:**
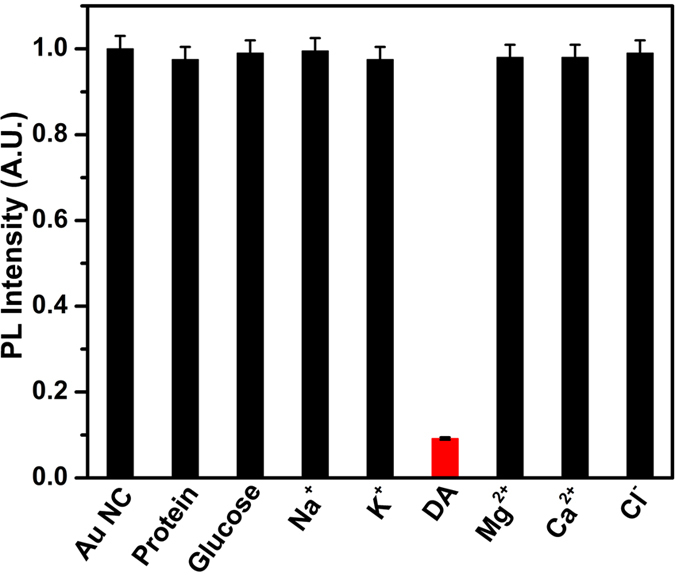
Comparison and selectivity of as-synthesized BSA-Au NCs for DA over the other molecules and ions. The concentration of all ions and molecules were used at 10 nM.

An excellent fluorescent probe should not only exhibit the high sensitivity, but also be enriched with specific selectivity. To evaluate the selectivity of this sensing system, we investigated the PL intensity changes of BSA-Au NCs in the presence of various molecules and metal ions which are naturally present in CSF under the same conditions and the results were presented in Fig. 10. It was demonstrated that a much lower (~91%) PL of BSA-Au NCs was observed upon addition of DA. In contrast, no significant decrease was observed by the addition of other molecules and ions the system. This observation clearly shows that our sensing platform was highly selective and sensitive towards the detection of DA in CSF.

#### PL quenching mechanism

**Figure 11:**
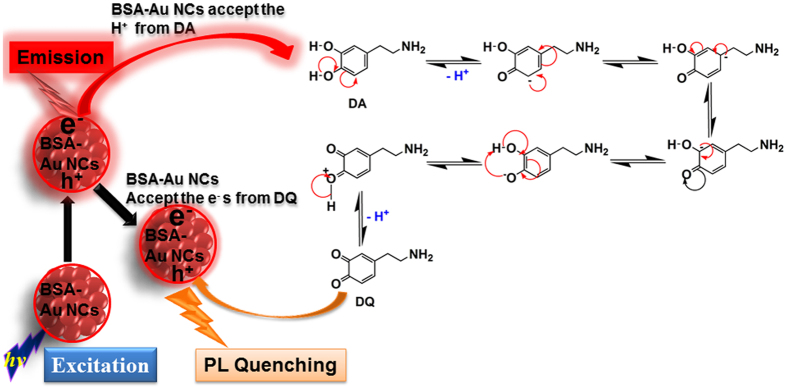
Schematic representation of fluorescence intensity quenching of BSA-Au NCs with DA and also figure shows the reaction mechanism of DA to form DQ via electrons transfers from DA to BSA-Au NCs and those electrons were donated to DQ leads fluorescence intensity quenching.

The possible mechanism of BSA-Au NCs fluorescence intensity quenching in the presence of dopamine is represented in Fig. 11. Dopamine has good ionizable properties due to the presence of two acidic protons, with the value of approximately ~9 pK^34^. In the presence of the suitable amount of photon energy (*hv*), BSA-Au NCs electrons (e^−^) were excited into the conduction band (CB) from the valence band (VB), eventually forming an exciton or electron-hole pair. These excited-state excitons of BSA-Au NCs readily reacted with dopamine, and abstracted those two acidic protons from dopamine. Dopamine also readily donated these two acidic protons to BSA-Au NCs, in order to form DQ via a stable phenoxide-enolate. This phenoxide-enolate was stable due to its resonance structures around the aromatic ring. The negative charge on the oxygen atom of dopamine is delocalized around the ring. One of the lone pairs on the oxygen atom overlaps with the delocalized electrons on the benzene ring. This overlap leads to a delocalization, which extends from the ring over the oxygen atom. Eventually, these electrons from the BSA-Au NCs were donated to DQ, leading to the quenching of BSA-Au NCs fluorescence intensity via the electron transfer mechanism.

#### Life time decay studies

**Figure 12:**
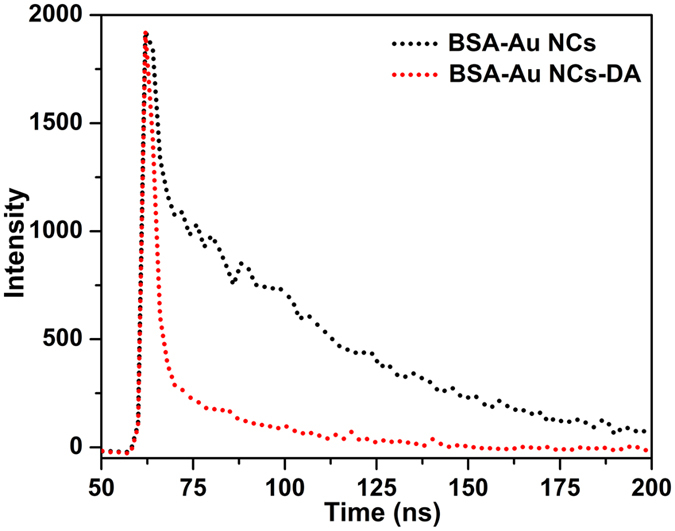
Time resolved fluorescence decay of BSA-Au NCsin absence and presence of DA.

The fluorescence quenching of as-synthesized Au-Cluster in presence of DA was determined by using time resolved fluorescence decay study as demonstrated in Fig. 12. The fluorescence decay of all curves can be fitted by a double exponential function by using following expression:


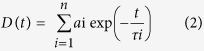


where D is PL decay, *τ* is life time, *τ*_*i*_ is PL lifetimes of various fluorescent forms and *a*_*i*_ is corresponding pre-exponential factors^35^. The fluorescence lifetime decay was estimated at 450 nm excitation wavelength using colloidal silica particle solution as a reference. The Au-Cluster shows the lifetime of *τ*_*1*_ 0.18, and *τ*_*2*_ 0.96 ns in the absence of the acceptor, whereas the lifetime of τ_1_ 0.01, and τ_2_ 0.1 ns was significantly decreased after addition of acceptor DA, which clearly indicates Au- NCs transfers the electrons to DQ and it acts as an energy acceptor to quench the fluorescence intensity of Au-Cluster intensity via the electron transfer mechanism.

## Conclusion

In this study, we demonstrated the synthesis of protein protected fluorescent gold clusters for the rapid, simple, and sensitive detection of DA in CSF. The resulting BSA-Au NCs particles were well dispersed, and were 4–6 nm in size, with ~8% of QY, strong fluorescence property, and good stability. The evolution was investigated using diverse tools, such as X-ray photoelectron spectroscopy (XPS), photoluminescence spectroscopy (PL), UV-vis spectroscopy, X-ray diffraction (XRD), and high-resolution transmission microscopy (HRTEM). BSA-Au NCs bind strongly with DA, and this provides the selective detection limit of 0.622 nM and capable to detect DA in biological body fluids such as CSF with high sensitivity and selectivity. This indicates that our approach may useful for the development of simple, less expensive, ultra-sensitive methods for the detection of DA, and may be beneficial in a wide range of nanotechnology applications and in the biosensor field.

## Additional Information

**How to cite this article**: Govindaraju, S. *et al*. Fluorescent Gold Nanoclusters for Selective Detection of Dopamine in Cerebrospinal fluid. *Sci. Rep.* **7**, 40298; doi: 10.1038/srep40298 (2017).

**Publisher's note:** Springer Nature remains neutral with regard to jurisdictional claims in published maps and institutional affiliations.

## Figures and Tables

**Table 1:** Detection performance of different fluorescent sensors for detection of dopamine.

Material	Technique	Limit of detection	Reference
Mesoporous nitrogen carbon materials	Electrochemical sensor	0.001 μM	36
d-DNA copper nanoparticles	Fluorescent sensor	20 pM	37
Nanoporous AuAg alloy	Electrochemical sensor	0.2 μM	38
Carbon dots and nano-graphite	Fluorescent sensor	0.055 nm	39
Carbon nanopipette	Electrochemical sensor	25 ± 5 nM	40
Gold nanoparticles	Colorimetric sensor	33 nM	41
Poly(tetrafluoroethylene)	Self-powered triboelectric nanosensors	0.5 μM	42
NH2–graphene	Electrochemiluminescence sensor	0.04 μM	43
CdSe quantum dots	Electrochemiluminescence sensor	0.5 μM	44
Pd/Bacterial Cellulose Hybrid Nanofibers	Electrochemical biosensor	1.26 μM	45
**BSA-Au NCs**	**Fluorescence quenching**	**0.622 nM**	**Present work**

**Table 2:** Detection of DA (nM) in spiked CSF.

Sample	Spiked	Found ± S.D	% Recovery	RSD (%)
**CSF**	1	1.05 ± 0.078	108.42	6.66
	2	2.15 ± 0.187	109.28	8.37
	4	3.94 ± 0.040	97.82	1.01
	6	5.68 ± 0.422	88.12	7.42
